# A Personalized Self-Management Rehabilitation System with an Intelligent Shoe for Stroke Survivors: A Realist Evaluation

**DOI:** 10.2196/rehab.5079

**Published:** 2016-01-07

**Authors:** Susan Mawson, Nasrin Nasr, Jack Parker, Richard Davies, Huiru Zheng, Gail Mountain

**Affiliations:** ^1^Rehabilitation and Assistive Technology Research GroupSchool of Health and Related ResearchUniversity of SheffieldSheffieldUnited Kingdom; ^2^Computer Science Research InstituteSchool of Computing and MathematicsUlster UniversityCounty AntrimUnited Kingdom

**Keywords:** stroke, self-management, design, realist evaluation, sensor technology, equipment design, telehealth, self-care

## Abstract

**Background:**

In the United Kingdom, stroke is the most significant cause of adult disability. Stroke survivors are frequently left with physical and psychological changes that can profoundly affect their functional ability, independence, and social participation. Research suggests that long-term, intense, task- and context-specific rehabilitation that is goal-oriented and environmentally enriched improves function, independence, and quality of life after a stroke. It is recommended that rehabilitation should continue until maximum recovery has been achieved. However, the increasing demand on services and financial constraints means that needs cannot be met through traditional face-to-face delivery of rehabilitation. Using a participatory design methodology, we developed an information communication technology–enhanced Personalized Self-Managed rehabilitation System (PSMrS) for stroke survivors with integrated insole sensor technology within an “intelligent shoe.”. The intervention model was based around a rehabilitation paradigm underpinned by theories of motor relearning and neuroplastic adaptation, motivational feedback, self-efficacy, and knowledge transfer.

**Objective:**

To understand the conditions under which this technology-based rehabilitation solution would most likely have an impact on the motor behavior of the user, what would work for whom, in what context, and how. We were interested in what aspects of the system would work best to facilitate the motor behavior change associated with self-managed rehabilitation and which user characteristics and circumstances of use could promote improved functional outcomes.

**Methods:**

We used a Realist Evaluation (RE) framework to evaluate the final prototype PSMrS with the assumption that the intervention consists of a series of configurations that include the Context of use, the underlying Mechanisms of change and the potential Outcomes or impacts (CMOs). We developed the CMOs from literature reviews and engagement with clinicians, users, and caregivers during a series of focus groups and home visits. These CMOs were then tested in five in-depth case studies with stroke survivors and their caregivers.

**Results:**

While two new propositions emerged, the second importantly related to the self-management aspects of the system. The study revealed that the system should also encourage independent use and the setting of personalized goals or activities.

**Conclusions:**

Information communication technology that purports to support the self-management of stroke rehabilitation should give significant consideration to the need for motivational feedback that provides quantitative, reliable, accurate, context-specific, and culturally sensitive information about the achievement of personalized goal-based activities.

## Introduction

In the United Kingdom, stroke is the most significant cause of adult disability. Stroke survivors are frequently left with physical and psychological changes that can profoundly affect their functional ability [1], independence [2], and social participation [3-6]. With the global incidence of stroke set to escalate from 15.3 million to 23 million by 2030 [7] and the decrease in mortality and rise in morbidity, more stroke survivors will be living with long-term disability [8].

Research suggests that long-term, intense, task-specific, context-specific, goal-oriented, variable rehabilitation that is goal-oriented and environmentally enriched improves function, independence, and quality of life after a stroke [9]. Over recent years, there has been a contextual shift in service delivery from hospital-based rehabilitation to the community. It is recommended that rehabilitation should continue until maximum recovery has been achieved [9,10]; however, the increasing demand on services and financial constraints mean that needs cannot be met through traditional face-to-face delivery of rehabilitation. Radical innovation and the adoption of a self-management paradigm need to be considered as a way to deliver home-based rehabilitation, thereby meeting the challenges faced in health care.

In 2007, the SMART consortium began a program of research to develop and evaluate an Information Communication Technology (ICT) enhanced Personalized Self-Managed System for people with complex long-term conditions [11,12]. The program aimed to deepen our understanding of the potential for technology to support self-management of long-term chronic conditions through an iterative, user-centered design methodology focused on health and social care [13]. Three conditions were chosen for the study—chronic pain, chronic heart failure, and stroke—with the intent of exploring how a multimodular system could support the three areas, with a proposition that other long-term conditions could be integrated into the system at a later stage. The intervention model for the stroke system was based around a rehabilitation paradigm underpinned by theories of motor relearning and neuroplastic adaptation, motivational feedback, self-efficacy, and knowledge transfer [14-17].

**Figure 1:**
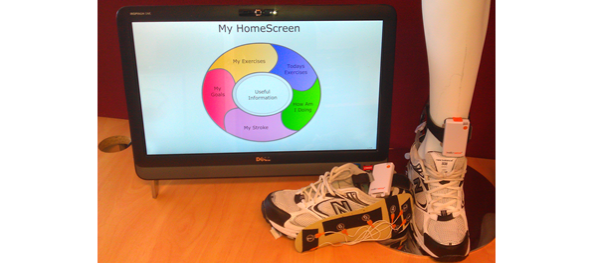
The PSMrS home hub for stroke survivors with insole and data logger providing walking feedback through the PSMrS.

**Figure 2:**
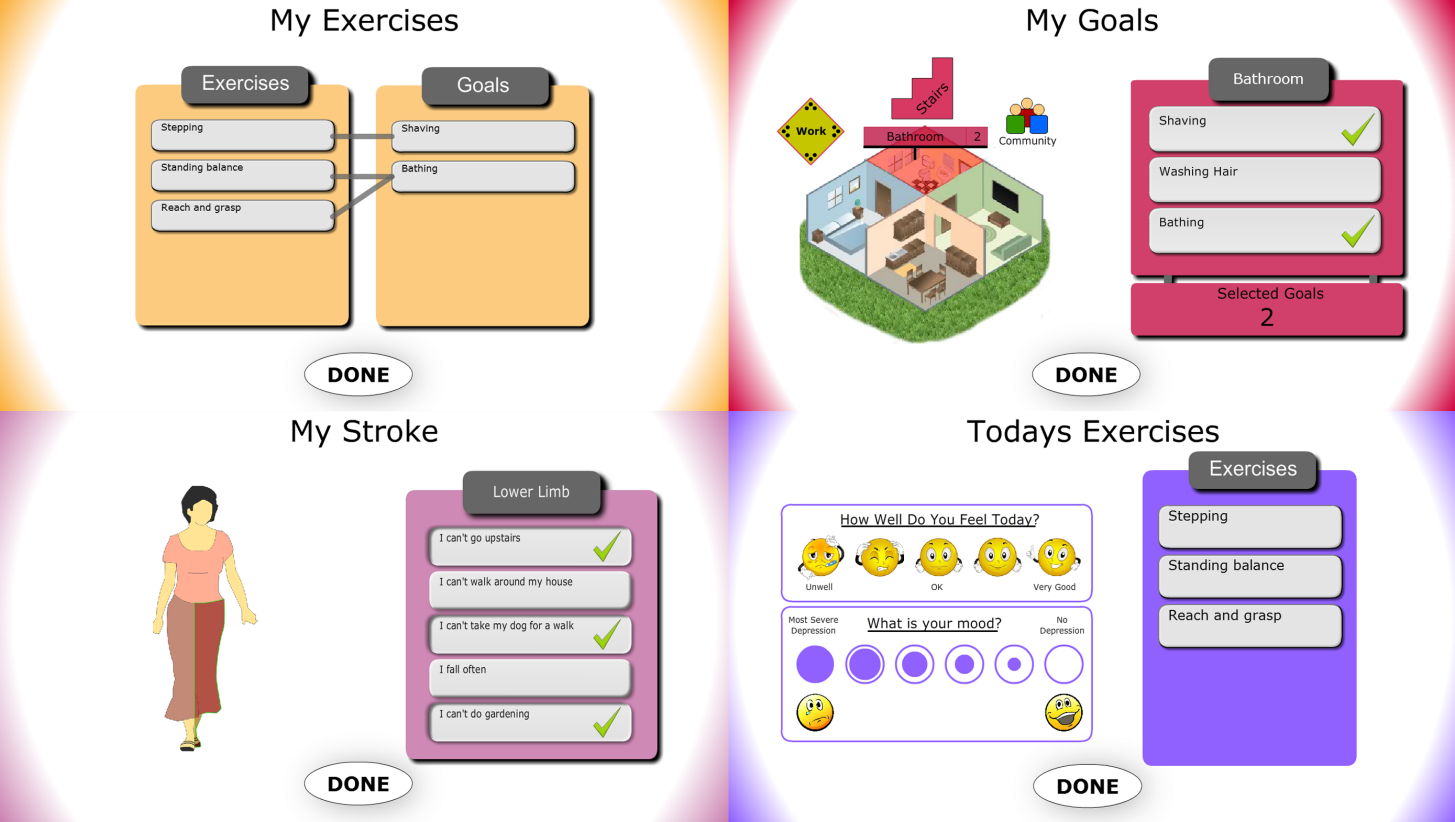
Screenshots of the PSMrS user interface.

**Figure 3:**
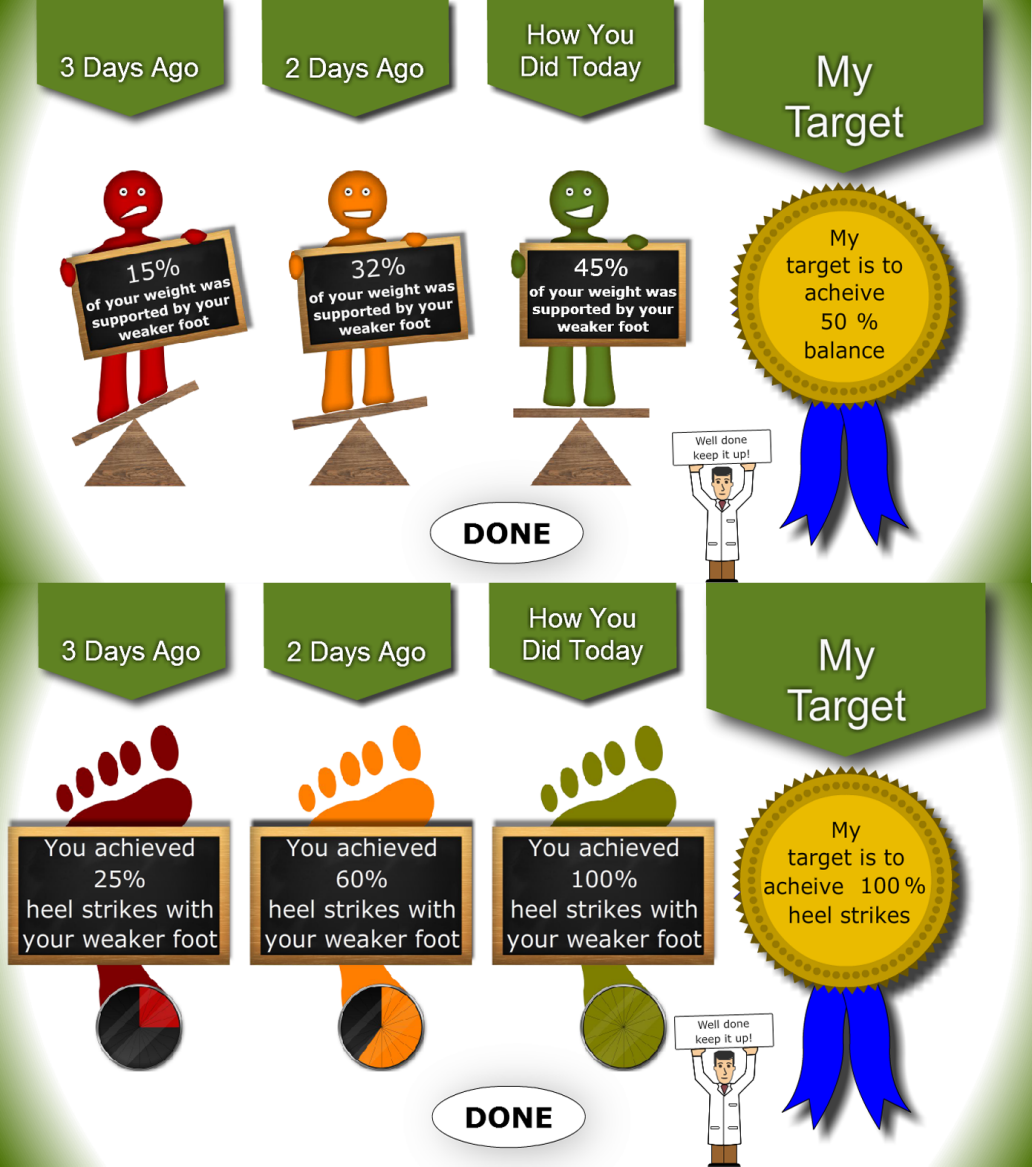
User feedback for symmetry and heel strike data from insole.

The SMART interdisciplinary research team applied a mix of health, social sciences, and user-centered design methods to develop the Personalized Self-Management Rehabilitation System (PSMrS) for stroke survivors [18]. The PSMrS is a prototype ICT system integrated with home hub sensor technology—the intelligent shoe—developed to enable stroke survivors to self-manage their rehabilitation to achieve identified life goals specific to them (Figures 1-3). While other wearable devices are available, the sensored insole was deemed to be the most appropriate as walking re-education and foot placement are key components of a stroke rehabilitation program. Data from the sensors give feedback to users through screens (Figure 3) designed with stroke survivors to depict balance and heel strike as a percentage of normal values. The aim of this final aspect of the research program was to understand the conditions under which this technology-based rehabilitation solution would most likely have an impact (outcome) on the motor behavior of the user, what would work for whom, in what context, and in what way.

## Methods

In order to enhance and strengthen our previous research, we used a Realist Evaluation (RE) approach [19] to evaluate the final prototype of PSMrS, prior to a feasibility pilot study, in order to explore in depth the value, usability, and potential impact such technology could have on an individual’s ability to self-manage their rehabilitation following a stroke.

Realist evaluation is a well-recognized methodology with its roots in philosophy, social sciences, and evaluation methods. To conduct realist evaluation, it is necessary to assume that the program (or in this case the PSMrS intervention) consists of a series of configurations that include the context, the underlying mechanisms of change, and the potential outcomes or impacts. Realist evaluation is underpinned by theory described as a set of prepositions about the nature of change that is predicted, as well as the hypothesis that change can be maintained by the action of particular mechanisms within particular contexts (eg, the proposition that a simple touch-screen computer interface can motivate people even with low or no computer literacy to use the system for monitoring their health in the context of their home).

**Figure 4:**
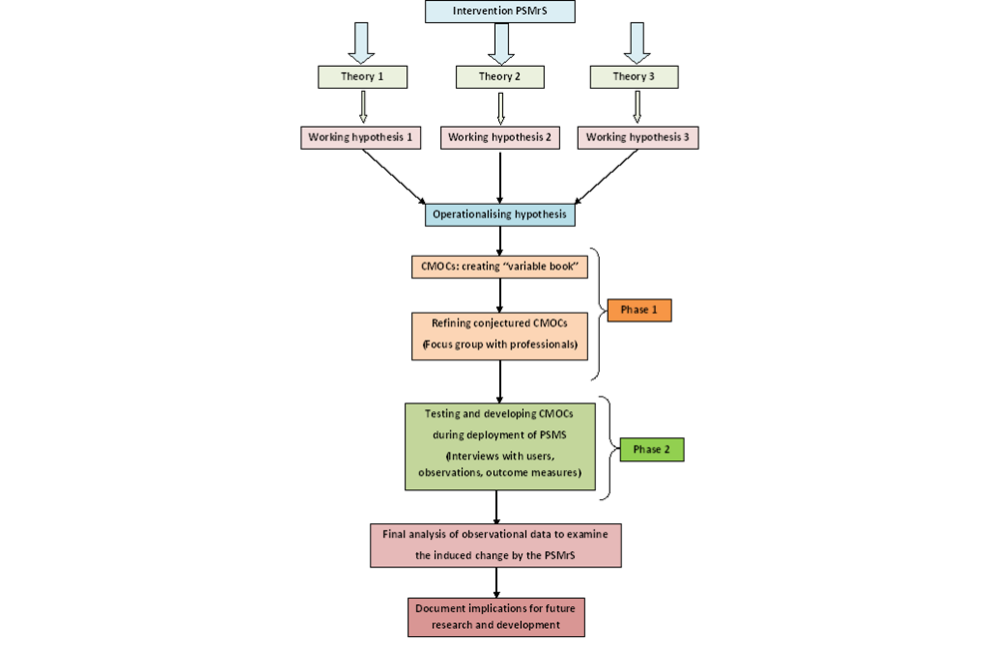
An overview of the realist evaluation plan.

This methodology also tries to explain those contexts that are “conducive” or “resistant” to change [20]. Any realist evaluation must fully engage stakeholders, clinicians, stroke survivors, and caregivers in the generation of theories to be tested through the evaluation and the identification of subsequent working hypotheses that then drive the evaluation process. An overview of the realist evaluation plan adopted in this research is summarized in Figure 4.

The overall evaluation questions for this research were what works, for whom, why, in what way, and under what circumstances? In the case of the PSMrS, we were interested in what aspects of the system would work best to facilitate the motor behavior change associated with self-managed rehabilitation and which stroke user characteristics and circumstances of use could promote improved functional outcomes.

The theories to be validated through the realist evaluation process were generated through literature reviews together with empirical data collected in the earlier work [18,21,22]. These theories were then validated or refuted through individual and focus group interviews conducted with patients/caregivers and health professionals as described below. There were a number of theories that we wished to explore in this aspect of the evaluation; for example, the theoretical models of self-management rehabilitation that are amenable to technological solutions, the implications of motor behavior change mechanisms such as neuroplasticity and how they can be taken into account in technology development, and the extent to which technology can facilitate a shift in responsibility for the management of care from the professional to the stroke survivor.

The theories generated a number of hypotheses/propositions, to be explored rather than tested:

1. Specific elements of self-management can be successfully promoted through the use of technology designed for this purpose.
2. This technology can help individuals relearn motor behavior by encouraging achievement of personal functional goals and repetition of key motor activities within those goals.
3. The technology facilitates partnership working between the user and others to achieve self-management.
4. The stroke PSMrS gives users the opportunity to perform exercises as much as they can through repetition and provides them with tailored feedback. Both these attributes can promote motor relearning and neuroplastic adaptation.
5. The technology can enable users to interpret physiological data through motivational feedback screens.
6. By mastering (mastery) the tasks involved in self-management programs and being provided evidence of this through real-time feedback on performance, users develop confidence (self-efficacy) that then leads to a more active role in the management of their condition.

In accordance with the realist evaluation methodology, the process of hypothesis validation and generation were followed by operationalization of the hypotheses into mechanism, context, and outcome configurations (CMOCs). These were explored, refined, developed, and tested through practitioner and participant engagement.

### Recruitment and Participant Involvement

All participants were recruited via health services and deemed to be fit to join the study by the referring physiotherapist. Ethics approval was obtained through the Leeds Ethics committee (08/H1306/46), and informed consent was obtained before the system was deployed to participants’ homes. All participants had to be able to comprehend written English, not have significant cognitive impairment, and be clinically stable. The stroke survivors needed to be willing and able to use the equipment and report back on their experiences to the research team. Specific inclusion criteria for participants were that they did not have any communication problems that would significantly impede comprehension or have severe hemiplegia to the extent that they were not able to get up out of the chair independently.

Participants’ demographic characteristics and baseline clinical data were recorded at the outset (see Table 1). The stroke survivors were also interviewed qualitatively before and after the period of installation about their views and experiences.

**Table 1:** Patient demographics.

Participant	Age of patient/ age of caregiver	Side affected	Time since stroke	Computer experience^a^	Able to comprehend written English	Walking aid
17	63/57	R hemi	13 months	++	Yes	None (FES)
23	73/73	L Hemi	18 months	+	Yes	Frame and tripod (FES)
33	45/44	R Hemi	18 months	+++	Yes	None (FES)
34	60/60	L Hemi	15 months	++	Yes	None (FES)
35	42/44	R Hemi	12 months	++	Yes	None (FES)

^a^+ denotes the amount of computer experience.

Information collected from the deployed systems were transferred and stored using a non-identifying format on a server hosted at one of the partner universities. The security and privacy of data between the stroke survivor’s devices and the server were protected using two methods. The first was to keep the data private by anonymizing all of the data so that sensitive information was never transmitted across the Internet. The second was to store the information in a secure manner; information was stored on a university server that was held in a secure room under lock and key and behind a firewall. In addition, the server was also active only during the realist evaluation and was disconnected from the Internet once the realist evaluation was completed. Technical support was available over the telephone and by researcher follow-up visits where necessary during office hours.

Five people with stroke were recruited from either Sheffield Community Intermediate Care Services or the Assessment and Rehabilitation Centre while they were still receiving rehabilitation. The engagement of therapists at this service was obtained through an initial focus group where the technology was explained and demonstrated together with the requirements for participant involvement (local agreement for access has already been obtained). Participants were identified during the period of community rehabilitation by the therapists, with the anticipation being that the PSMrS would be integrated into the standard stroke care pathway prior to discharge from the stroke service or where they were still engaged in active rehabilitation. The treating physiotherapist in partnership with the stroke survivor personalized the system and the stroke survivor (with or without their carer) practised using the system under supervision within the rehabilitation center. The service participants were then encouraged to continue using the system for up to 4 weeks independently at home. The participants were advised to contact a health care professional if any health issues arose during the deployment period. A researcher was available by telephone if technical difficulties arose during the 4-week period.

### Conducive Context

In order for the mechanisms underpinning the PSMrS to work, a number of generic contextual conditions had been previously identified [21,23]. The system had to be reliable, accurate, and robust; be adapted and personalized to the individual personal, environmental, and social context of the stroke survivor; be accessible in the home setting; be person-centered (customized for the individual) and used independently of the therapist; and provide the user with adequate resources to enable them to understand and have knowledge about their stroke and rehabilitation processes.

Examples of what some of the contexts, mechanisms, and outcomes (CMO) for the PSMrS are provided in Table 2. This combination of theory, hypothesis generation, and development of CMOC was the foundation work for the evaluation; a realist evaluation demands a synthesis of qualitative and quantitative methodology.

**Table 2:** Context mechanisms outcome configurations for the PSMrS.

Some plausible mechanisms (why)	Some potential contexts (who/in what circumstances)	Some possible outcomes
M1: By using the PSMrS, users will gain a sense of task mastery which might increase their confidence.	C1: A system that provides rewarding feedback as a result of improved symmetry and heel strikes.	O1: Increased confidence in the user’s ability to carry out everyday tasks. Measure: Qualitative data
M2: By using the PSMrS, users will be facilitated to set specific, measurable, attainable, realistic, and time-specified goals that might promote more responsibility towards their rehabilitation.	C2: A system that is used by a participant where they continue to desire improvement(s) and those improvements are achievable.	O2: Increased self-efficacy and ownership of their rehabilitation. Measure: Qualitative data
M3: By using the PSMrS, users performing selected exercises in the home and repeating these exercises might lead to users’ developing knowledge about carrying out stroke rehabilitation in the home environment.	C3, C3a: A system that can be used in the home and has specific goals and exercises that can be carried out within the home/domestic environment.	O3: Context-dependent/ place-based and culturally meaningful rehabilitation. Measure: Qualitative data and quantitative data from the TELER quiz style indicator.
M4: By using the PSMrS, users have problem-solving opportunities that might lead to the successful achievement of goals and attribution of success to users’ personal abilities.	C4: A system that enables users to set and achieve personal goals through shared decision-making between patients and professionals.	O4: Increased users’ agency and their active roles in self-management. Measure: Online data sources from insole and qualitative data.
M5: The use of the PSMrS will facilitate the translation of physiological data, which might enable the user to interpret their symptoms.	C5: A system that translates physiological data through feedback.	O5: An understanding of symptoms and change in symptoms throughout the usage of the system. Measure: Qualitative data and quantitative online data sources from insole.
M6: The use of the PSMrS might encourage increased intensity of practice with consequential neuroplastic changes.	C6: A system that provides individualized motivational feedback on the achievement of walking skill.	O6: Increased functioning and achievement of improved walking skill. Measure: Online quantitative data sources from insole.

### Observation of Context, Mechanism, and Outcome Configuration

Our goal was to gather both qualitative and quantitative data before, during, and after participant interaction with the technology. The quantitative data gathered before, during, and after the technology deployment enabled us to observe changes in physical activity, specifically walking ability, and quality and changes in knowledge levels. To achieve the latter, a measure called TELER Quiz style outcome indicators was used [24-26]. Quantitative walking data on heel strike, gait speed, and symmetry was recorded online from the sensors in the intelligent shoe during the time stroke survivors used the PSMrS. The amount of walking activity was also measured in order to provide data to support the proposition around neuroplastic adaption and intensity of practice. We also applied a measure of technology usability, the System Usability Scale (SUS) [27].

In order to ensure that the quantitative gait data gathered from the insole was valid, providing accurate and reliable results, two approaches were adopted. In the first instance, the hardware and sensor technology consisted entirely of off-the-shelf products that were then integrated into the PSMrS in a novel way. This ensured that the technology complied with European Union safety, health, and environmental requirements. In addition, there were assurances that the manufacture has produced a product that was fit for purpose and had been through rigorous manufacturing processes such as quality assurance and testing. Second, a consistent hardware configuration was adopted in relation to sensor deployment, as any deviation from this template would have serious implications on accuracy and repeatability of results.

Finally, qualitative and quantitative analysis was carried out across all participant cases to establish whether the theories underpinning the personalized self-management system had been supported or refuted [28] and to what extent the intervention had created change in user behavior. Due to the extensive amount of information gathered during the evaluation, this paper reports only the qualitative data with the quantitative data reported elsewhere [29].

### Data Analysis

The focus of the qualitative analysis was based on both the exploration of the pre-existing context and the development and refinement of the hypothesized CMOC using thematic analysis [30-32]. This innovative approach to the analysis draws on Yin [30], Miles and Huberman [33], and Patton [34] and is underpinned by the principles of realist evaluation [19].

This approach allowed for themes to emerge from the data and examines interconnections and relationships between the mechanisms and contexts in relation to proposed outcomes [31,32,35].

## Results

The next stage of the realist evaluation cycle (see Figure 3) involves the specification phase where findings are synthesized and presented as refined CMO configurations to answer the question, “What works for whom and in what circumstances and ways?” [19,20].

### What Work Works for Whom, and in What Circumstances and Ways?

Data analysis reveals that in order to achieve desired outcomes through the use of computer technology, a number of issues—such as the technology itself, the provision of feedback, the motivation of the user and what impacts on this, and the personal and social environment in which the system is used—can affect the mechanisms underpinning the intervention. The following section will discuss each of these issues in detail.

### Technology

The limitations of PSMrS and the SMART insole had an impact on the usage of the system. Users relied on their caregiver to don the anklet, three of the five experienced Internet connection difficulties, the system required re-booting due to freezes, and the on/off switch was fragile and subsequently needed replacing: “It’s quite fiddly to get the devices around the ankles and the insoles could do with being stiffer” (Participant 17) and “I always set off on my walking with my heart in my mouth thinking ‘is it going to work?’!” (Participant 23).

Due to storage and accessibility issues, 3 users suggested that they would have preferred alternative devices to view their feedback such as a tablet or smartphone: “that [PSMrS] is a little bit cumbersome…if that could have been a laptop or an iPad size where you could put it somewhere. You could hold it on your knee” (Participant 35).

### Feedback

Receiving feedback following performance was of particular importance to the users. More specifically, the provision of accurate, reliable quantitative Knowledge of Results (KR) feedback of goal attainment (ie, 100% heel strikes) affected users’ motivation to use the system: “Having a numerical result to what you’re doing helps because it is very easy to see that you’ve got an improvement” (Participant 23).

All of the users described how being able to make visible the invisible, observe their improvement, and track progress over time was of great importance. This would not only indicate that they are continuing to make improvements but they are also “returning to normality.” They were therefore using improved scores as recovery markers: “It makes me feel like I’m making progress. I’m going down that road to full recovery. I know full recovery is never going to happen but I just keep saying to J I’ve passed another milestone” (Participant 23).

However, trusting the PSMrS and the scores provided affected their usage. For example, one of the users suggested that the system provided unexpected results: “you might not walk perfectly but the machine says that you’re doing quite well!” (Participant 17).

Interestingly, 2 users reported practicing walking around without the SMART insole in their shoes so that when they used the insoles, they might get a better score: “I got it down in the low thirties…so without the sensors on we did an exaggerated heel–toe, the next time the score had improved a lot” (Participant 23).

### Motivation

Motivation emerged as being related to feedback in that the scores obtained following performance focused their determination to improve. The users expressed their desire to strive for better scores following feedback: “I shouldn’t be satisfied until I’m in the green and that little man pops up” (Participant 23).

Notably, because they had a score for their performance, the users were able to involve significant others, which reinforced behavioral change. This would involve caregivers and family members expressing their admiration for the improvements made, which would instill a level of mastery and confidence.

Researchers were interested in the consequences of negative feedback, that is, how they would respond if they received a poorer score than previously achieved. However, all of the users suggested it increased their determination: “It made me want to do it again, to better it!” (Participant 35).

However, a number of negative factors affected the motivation, such as fear of failure (users would practice without the shoe to ensure they achieved a better score) and self-awareness of their limitations (they were aware of how far they could walk, the risk of falling, environmental obstacles, fatigue, and the concerns of caregivers/family members).

Furthermore, the caregivers also influenced user motivation. Caregivers had safety concerns that the stroke survivor would push themselves too far in an attempt to achieve greater scores: “I’m getting more relaxed with it than I was when I thought b****y hell, what’s she doing!!” (Caregiver 35).

### Self-Management

A number of self-management principles were observed during testing. These included problem-solving whereby users would make a conscious effort to change their movements to obtain higher scores, promoting self-efficacy through mastery, involving others in the process of rehabilitation to reinforce behavior change, and utilizing resources (using the system and its components to improve): “It makes me feel like I’m making progress. I’m going down that road to full recovery” (Participant 23) and “Oh I’m confident yes, yes! Just little things like in a morning when I’m at the wash basin in the bathroom I do free standing now as a matter of course” (Participant 23).

Two users described how close family members noticed their improvements, which provided encouragement and reinforced their efforts to continue striving for improvements. Participant 35 described how she was able to open the door for her grandchildren when they had come to visit:

My nanna look at my nanna!” And it’s what I used to do whenever they used to come. I used to go to the door and open the door for them. And I’d done it again, hadn’t I? And he [son] said it really did them good to see you do that!

Participant 35

Occasionally [granddaughter] says to me that I’m getting like the grandma that I used to be…she tells me know that I’m getting back to where I was.

Participant 35

### Context, Mechanism, and Outcome Configuration Refinement

This research aimed to test and refine intervention theories by exploring the complex interactions of contexts, mechanisms, and outcomes. Table 3 sets out the refinement of pre-existing CMOCs and highlights the changes following the observation of these CMOCs.

**Table 3:** Refinement of CMOC following observations and analysis.

Some plausible mechanisms (why)	Some potential contexts (who/in what circumstances)	Some possible outcomes
M1: By using the PSMrS, users will gain a sense of task mastery which might increase their confidence.	C1: A system that provides rewarding feedback as a result of improved symmetry and heel strikes.	O1: Increased confidence in the user’s ability to carry out everyday tasks. Measure: Qualitative data
M2: By using the PSMrS, users will be facilitated to set specific, measurable, attainable, realistic, and time-specified goals that might promote more responsibility towards their rehabilitation.	C2: A system that is used by a participant where they continue to desire improvement(s) and those improvements are achievable and that provides accurate, reliable, quantitative KR feedback of goal attainment.	O2: Increased self-efficacy and ownership of their rehabilitation. Measure: qualitative data
M3: By using the PSMrS, users performing selected exercises in the home and repeating these exercises might lead to users developing knowledge about the importance of carrying out stroke rehabilitation in the home environment for recovery.	C3: A system that can be used in the home and has specific goals and exercises that can be carried out within the context of the home/domestic environment and provides meaningful feedback following goal-based activity; C3a: A system that can be used in the home and has specific goals and exercises that can be carried out within the context of the home/domestic environment.	O3: Context-dependent/place-based and culturally meaningful rehabilitation. Measure: qualitative data; O3a: An awareness of the need to carry out rehabilitation
M4: By using the PSMrS, users have problem-solving opportunities that might lead to the successful achievement of activities/goals and attribution of success to users’ personal abilities.	C4: A system that enables users to set and achieve personal goals through shared decision-making between patients and professionals; C4a: A system that encourages independent use in the home and to set personal goals.	O4: Increased users’ agency and their active roles in self-management taking action (practicing). Measure: Online data date sourced from insole; Qualitative data
M5: The use of the PSMrS will facilitate the translation of physiological data, which might enable users to interpret their symptoms.	C5: A system that translates physiological data through feedback.	O5: An understanding of symptoms and change in symptoms throughout the usage of the system. Measure: Qualitative data; online data sources from insole.
M6: The use of the PSMrS might encourage increased intensity of practice with consequential neuroplastic changes.	C6: A system that provides individualized accurate, reliable quantitative motivational feedback on the achievement of specific tasks.	O6: Increased functioning and achievement of life goals. Measure: TELER, online data sources from insole.

## Discussion

### Principal Findings

This realist evaluation set out to explore the conditions under which this technology-based rehabilitation solution would most likely have an impact (outcome) on the motor behavior of people with stroke, what would work for whom, within a home context, and in what ways the system would have an impact. The pre-existing CMOs were based on theories of motor relearning, neuroplastic adaptation, and behavior change, specifically on the theories underpinning self-efficacy and the relationship between changes in self-efficacy and self-managed behaviors. The findings of the study confirmed the original CMOs and further highlighted two emerging propositions related to the context of use together with two new outcomes that were recorded in the qualitative transcripts.

The first proposition, which is perhaps to be expected, relates to the need for the system to be reliable and accurate in terms of providing quantitative feedback to the stroke users. The results suggest that this feedback should be about the attainment of goal-based activities with a specific emphasis on “knowledge of results.” The second proposition to emerge was related to the self-management aspects of the system. The study revealed that the system should also encourage independent use and the setting of personalized goals or activities. The stroke survivors identified the importance of goals using the words “activities” and “goals” interchangeably.

The outcomes identified from the data were first related to the users’ agency and their active role in self-management, where it emerged that “taking action” independently was an important outcome. The second related to “knowledge gain” where users became aware of the need to carry out rehabilitation in order to achieve their identified goal. This finding links well to the pre-existing CMO where the need for context-dependant and culturally meaningful rehabilitation had been identified as an outcome.

We suggest two implications that this study may have for both clinical practice and research. First the findings suggest any system that purports to support the self-management of stroke rehabilitation should give significant consideration to the need for motivational feedback that provides quantitative, reliable, accurate, context-specific, and culturally sensitive information about the achievement of personalized goal-based activities. A second implication is the role that complex interventions such as the PSMrS could have in changing knowledge and attitude to lead to behavior change. The PSMrS is a systems change intervention with complex effects in which contextual factors such as a network of relationships, as illustrated in this study, play a significant role in how the intervention is used and how sets of interdependent factors affect an individual’s decision to use the system [36].

### Conclusions

The research consortium will take this confirmation of theory and development of new propositions and recommendations into the development of the next iteration of the system prior to the implementation of robust population-based evaluation of a defined technology. This will test the effectiveness of the system in the promotion of self-managed rehabilitation and recovery.

In its current form, the system and in particular all of its software components are available to be deployed on a personal computer and smartphone. Current trends within computing indicate that the adoption of mobile computing continues to grow and dominate the market place. Therefore, plans for future work would focus on porting the current system to mobile-only platforms such as tablets and mobile phones. There are a number of advantages to doing this. Usability can be improved as mobile devices offer more flexibility and can operate in a wide range of environments and scenarios. Furthermore, practical considerations relating to the management and operation of any future randomized controlled trial would be more easily controlled.

## Abbreviations

CMO: contexts, mechanisms, and outcomes
CMOC: mechanism, context, and outcome configurations
FES: functional electrical stimulation
ICT: information communication technology
KR: knowledge of results
PSMrS: Personalized Self-Management Rehabilitation System
RE: realist evaluation
SUS: System Usability Scale
TELER: system for making and presenting clinical notes on a patient so that they can be used to establish the effectiveness of the treatment or care
